# TUBGCP2 variants cause lissencephaly spectrum disorders: a case report and literature review

**DOI:** 10.3389/fped.2025.1476390

**Published:** 2025-02-13

**Authors:** Tao Yu, Miao Yu, Xueyan Liu, Hua Wang

**Affiliations:** Department of Pediatric Neurology, Shengjing Hospital of China Medical University, Shenyang, China

**Keywords:** TUBGCP2, lissencephaly, genetics, neurodevelopmental disorder, microcephaly

## Abstract

**Background:**

TUBGCP2 variants are associated with the LIS spectrum disorders, but its pathogenesis remains unclear. To retrospectively analyze the clinical features and genetic information of patients having lissencephaly spectrum disorders associated with TUBGCP2 variants.

**Methods:**

Clinical and genetic data of a patient diagnosed with TUBGCP2-related lissencephaly spectrum disorder at the Department of Pediatric Neurology, Shengjing Hospital, in March 2022 were collected. Furthermore, we reviewed previously reported literature on patients with the same gene variation.

**Results:**

A 6-year-old female patient presented with microcephaly (head circumference: 46 cm, Z score: <−3), narrow forehead, thick eyebrows, bulbous nose, smooth philtrum, widened and separated teeth, speech and motor developmental delay, intellectual disability, and seizures. Brain magnetic resonance imaging showed pachygyria in the temporal, parietal, and occipital lobes. Gene testing identified hemizygous variation in TUBGCP2 (missense variants: c.178 C>T, c.538T>C, and maternal exon variant: 2–14 deletion). A literature search revealed seven patients with lissencephaly spectrum disorders associated with TUBGCP2 variants, including eight gene variation types. Moreover, the TUBGCP2 variants were found to cause lissencephaly spectrum diseases, with the main clinical manifestations being microcephaly, lissencephaly (including agyria, pachygyria, or subcortical band heterotopia), dysmorphic facial features (e.g., narrow forehead, thick eyebrows, bulbous nose, prominent ears, and widened and separated teeth), and developmental delay, with or without seizures.

**Conclusion:**

Our study expands the genotype of this brain malformation disorder associated with TUBGCP2 variants by presenting the first case of TUBGCP2 variants causing lissencephaly spectrum disorders in China.

## 1 Introduction

Lissencephaly (LIS) comprises a spectrum of cortical development malformations, including agyria, pachygyria, and subcortical band heterotopia (SBH) (1). The main pathological features of LIS are macrogyria, shallow sulci, and a thick cortex. The affected patients often present clinically with developmental delay (DD), intellectual disability (ID), and seizures. LIS can be caused by varied etiologies, such as toxicant exposure during pregnancy, fetal viral infections, and genetic or chromosomal abnormalities. Moreover, the increasing application of genetic screening in clinical settings has led to a notable rise in the number of identified LIS-associated genes. The expanded genetic testing has further revealed significant genetic heterogeneity in this condition, with most detected genes encoding proteins related to microtubules (MTs) or MT function. Currently, more than 20 LIS-related genes have been reported, including LIS1, DCX, tubulin gene, ARX, and Reelin pathway-related genes. The LIS spectrum disorders associated with bi-allelic pathogenic variants in TUBGCP2 were first discovered by Mitani et al. in 2019 (2).

In this study, we reported a 6-year-old patient with LIS caused by a hemizygous variant in TUBGCP2 (two paternal missense variants: c.178 C>T, c.538T>C and one maternal exon variant: 2–14 deletion). This study is the first reported case of a patient with the TUBGCP2 variant in China, thereby extending the genotypic spectrum of TUBGCP2-associated LIS. Additionally, we reviewed the previously published literature on patients with the same gene variation, aiming to provide physicians with an understanding of the clinical phenotype and genetics of this rare disease.

## 2 Materials and methods

### 2.1 Patient

The following basic information and clinical data of the patient was collected, including age, gender, age of onset, birth history, history of growth and development, family history, neurological signs, and the results of the laboratory tests, brain magnetic resonance imaging (MRI), electroencephalogram (EEG), and genetic test. This study was approved by the Ethics Committee of the Shengjing Hospital of China Medical University. The patient's legal guardians provided signed informed consent for this study.

### 2.2 Genetic variation analysis

DNA was obtained from the peripheral venous blood samples of the patient and the parents and submitted to the Chigene Translational Medicine Research Center Co., Ltd., Beijing, for trio-whole exome sequencing (WES) of the parents and proband. Initially, whole-exome capture was performed using an xGen Exome Research Panel v2.0 (IDT, Iowa, United States). The sequencing operation flow was then standardized on the DNBSEQ-T7 platform (BGI, China), followed by processing of the raw-sequencing reads via fastp (https://github.com/OpenGene/fastp) for adapter removal and filtering low-quality reads. Next, high-quality sequencing data were generated and mapped to the Ensemble GRCh37/hg19 reference genome using the Burrows–Wheeler Aligner (https://github.com/lh3/bwa). GATK (http://www.broadinstitute.org/gatk/) was further employed to recalibrate the base quality score and call SNP and INDEL variants. Trio-WES had a mean coverage depth of at least 172× per sample, with 97% of the exome covered 20× or greater. The sequencing depth ranged from 192× to 413× coverage of the TUBGCP2 gene, with 100% target region coverage >10× of the sequencing depth. Pathogenicity of the genetic variants was predicted using bioinformatics tools, such as PolyPhen (http://www.bork.embl-heidelberg.de/PolyPhen/), MutationTaster (http://www.mutationtaster.org), REVEL (https://sites.google.com/site/revelgenomics/), and CADD (http://cadd.gs.washington.edu/). Finally, all variants were classified according to the American College of Medical Genetics and Genomics (ACMG) guidelines (3).

The pathogenic variants detected using trio-WES were further validated by Sanger sequencing and qPCR. Primer 5.0 software was utilized to design the TUBGCP2 primers. Sanger sequencing was then performed using the 3730xl DNA Analyzer (Applied Biosystems, United States). Additionally, we designed specific fluorescent quantitative primers of the *TUBGCP2* exons 2, 8, and 14. Amplifications were conducted using qPCR, with the albumin gene as the reference gene. All the primers applied in Sanger sequencing and qPCR are listed in Supplementary Tables S1 and S2. The identified rare variants were submitted to the ClinVar database (https://www.ncbi.nlm.nih.gov/clinvar/; accession number: SCV002055968).

The TUBGCP2 protein is highly conserved among vertebrates, demonstrating 86% sequence identity among 10 different species, including primates, rodents, laurasiatheria, placental mammals, sauropsida, and fish (http://asia.ensembl.org/index.html). Furthermore, multiple protein sequence alignments were constructed on MEGA X (4), followed by 3D modeling of the structural effects based on the TUBGCP2 protein structure (AlphaFold, AF-P48058-F1) (5). The generated models were visualized in PyMOL (https://www.pymol.org) (6).

### 2.3 Literature review

**Figure 1:**
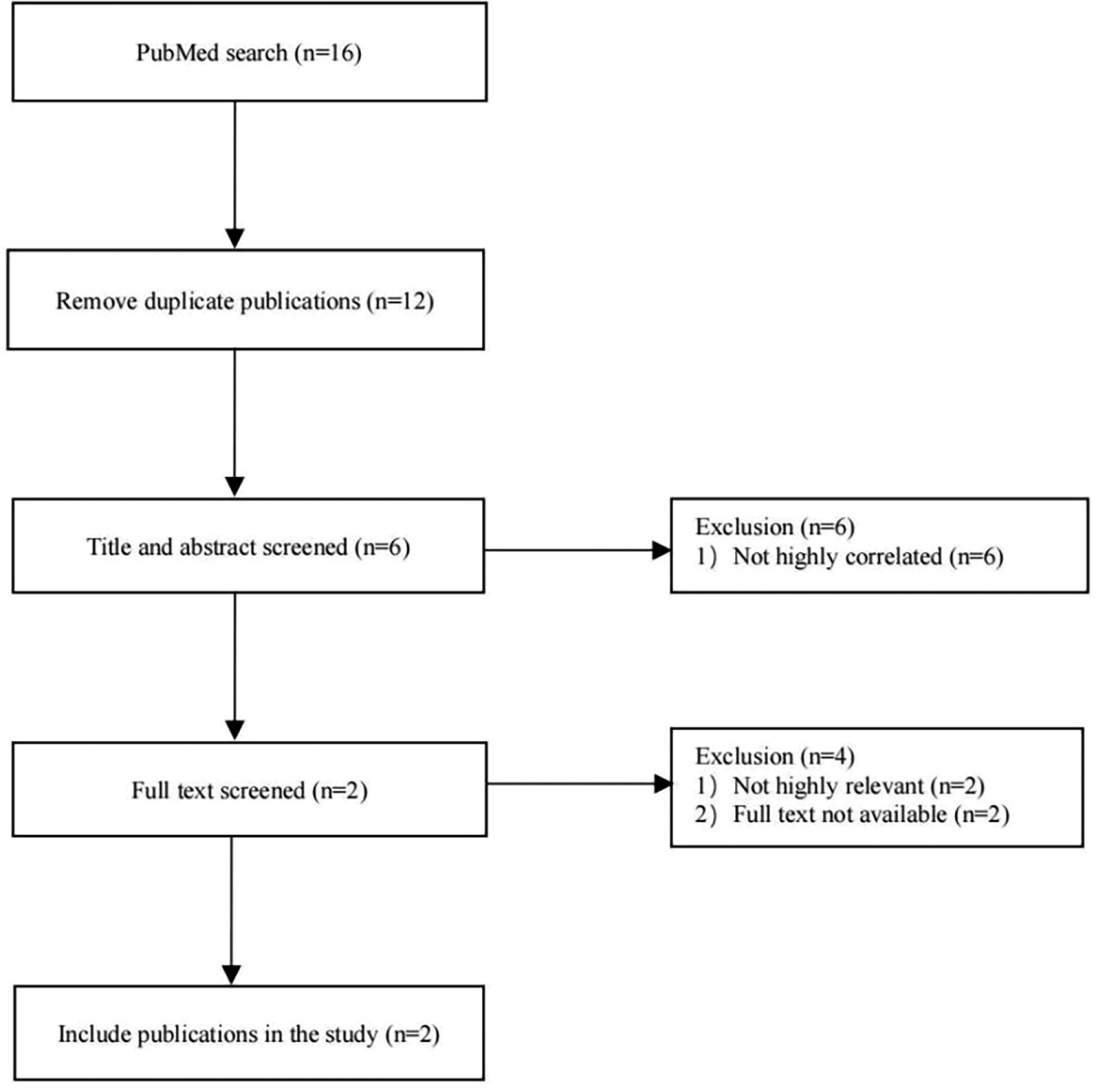
Flow chart of literature search and screening.

PubMed was searched for case reports published before September 01, 2023, using the search terms “TUBGCP2 mutation or variants in TUBGCP2,” “lissencephaly,” and “microcephaly”. (Figure 1) Moreover, case reports including patients with no or little clinical information were excluded from the review. Detailed information was collected from each case report using a pre-designed form, encompassing general demographic data, clinical manifestations, EEG and MRI findings, and TUBGCP2 variant details.

## 3 Results

### 3.1 Case report

**Figure 2:**
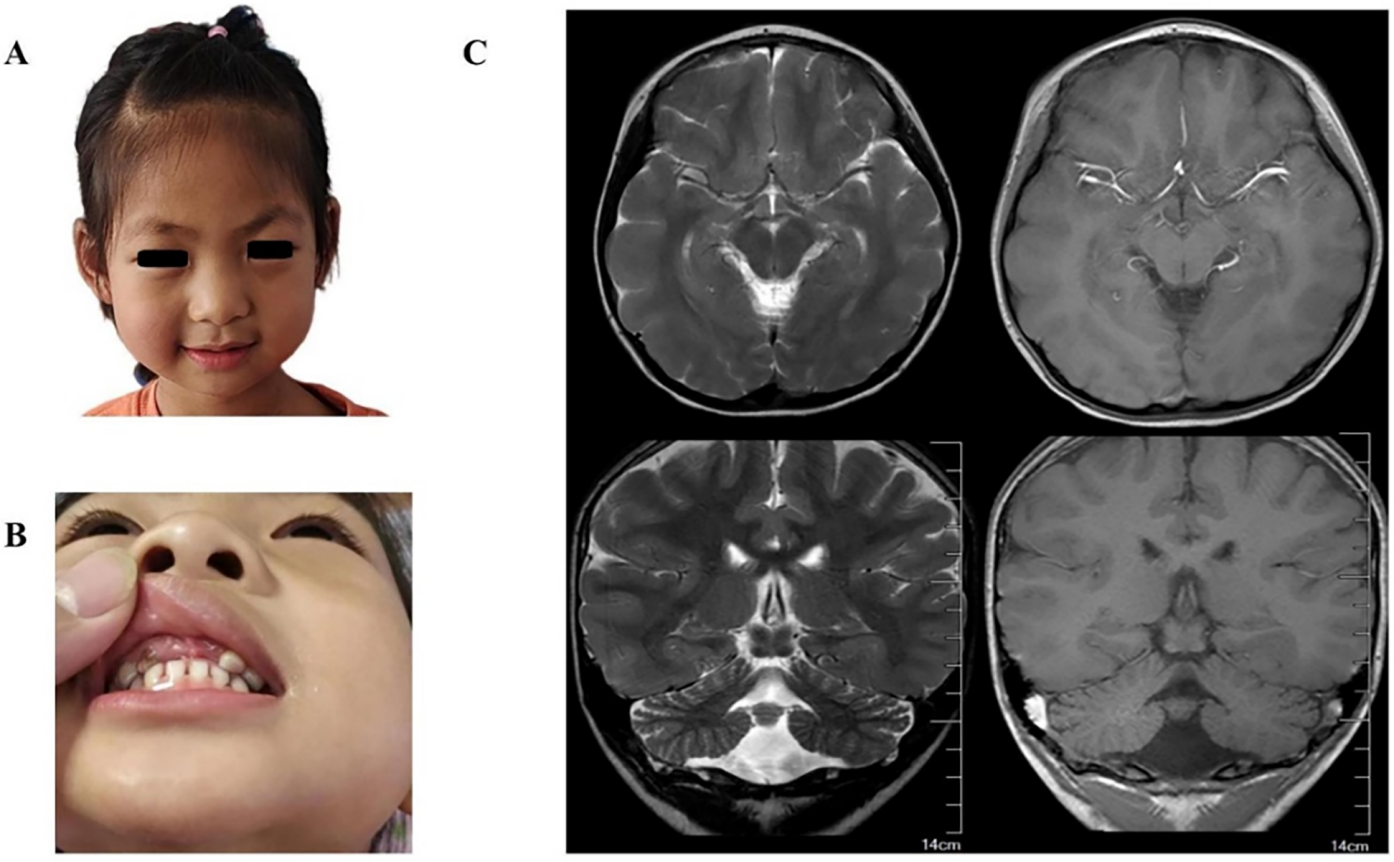
**(A)** photograph of the patient at 6 years showed narrow forehead, thick eyebrows, bulbous nose, smooth philtrum. **(B)** Photograph of the patient showed widened and separated teeth. **(C)** Brain MR images showed temporal, parietal and occipital pachygyria (the right is more prominent).

**Figure 3:**
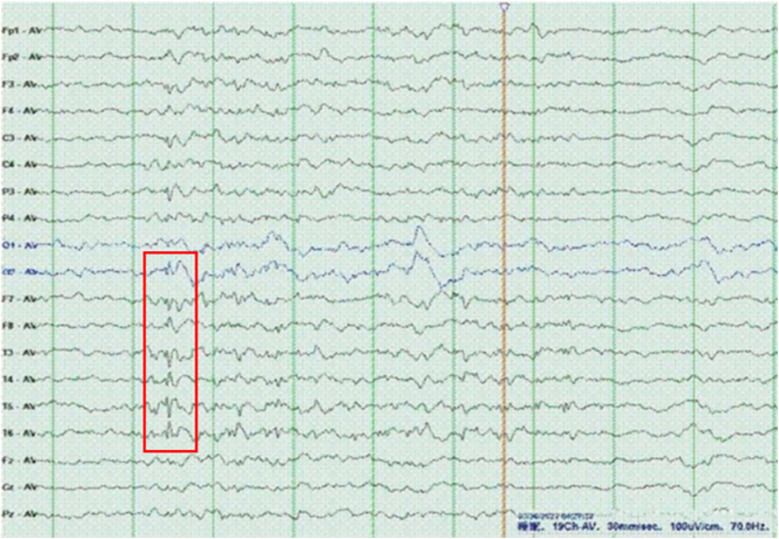
EEG of the patient revealed sharp slow waves and spike slow waves. EEG parameters: Reading paper speed: 30 mm/s; sensitivity: 10μV/mm; HF (high-frequenc.filters): 70 Hz; LF (low-frequenc.filters): 1 Hz; notch filter: 50H.

The proband was a 6-year-old girl who was the first child of healthy Chinese parents. The mother and father were aged 31 years and 33 years, respectively. The child was hospitalized due to unprovoked convulsions. Clinical manifestations comprised microcephaly (head circumference: 46 cm, Z score: <−3), narrow forehead, thick eyebrows, bulbous nose, smooth philtrum, widened and separated teeth, speech and motor developmental delay, intellectual disability, and seizures (Figures 2A,B). The child was delivered without complications at term, exhibiting no hypoxia or asphyxia. However, the patient's head circumference was not recorded at birth. Developmental stages were achieved as follows: head raising at 3 months, sitting at 10 months, crawling and standing at 12 months, and independent walking at 2 years. Furthermore, the child showed speech delay and could speak single words at 18 months. Currently, the patient can only express short sentences of five or six words. Moreover, the child experienced febrile convulsions at 2.5, 3.5, 4, and 5 years of age. On admission, physical examination revealed that the child's head circumference was only 46 cm (Z score <−3). Additionally, brain MRI (Figure 2C) indicated pachygyria in the temporal, parietal, and occipital lobes, while laboratory tests detected no abnormalities. EEG images displayed scattered spike slow waves and sharp slow waves in the posterior head region (Figure 3).

**Figure 4:**
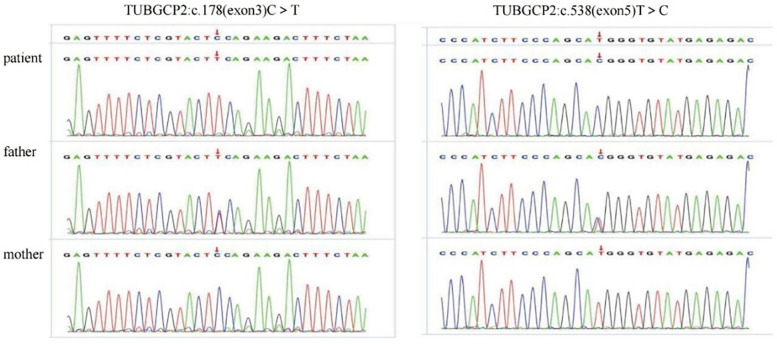
Sanger sequencing validation of the variants c.178 C >T and c.538T > C in the patient and parents.

**Figure 5:**
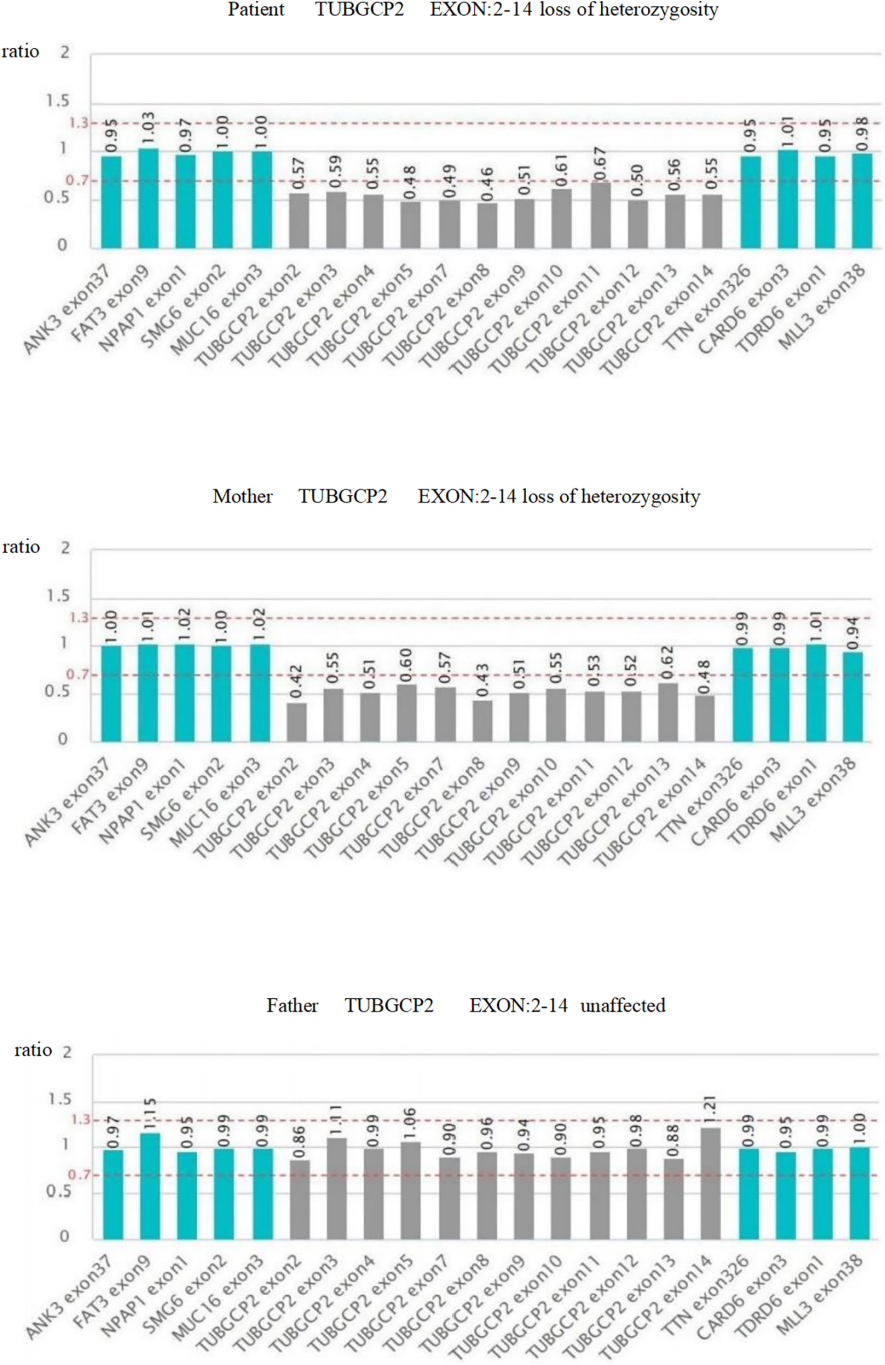
Schematic diagram of the exon deletion/repetition.

Trio-WES identified three TUBGCP2 variants in the patient: chr10:135111534-c.538 (exon: 5) T>C, chr10:13511359° c.178 (exon: 3) C>T, and chr10:135098588-135116445-loss1 (exon: 2–14) (Figures 4, 5). These variants were associated with disease features such as pachygyria, microcephaly, DD, and dysmorphic facial features, with or without seizures. According to the ACMG guidelines, the judgment basis for the three variants was as follows: PVS1 + PM2, PM2 + PM3 + PP3 + PP4, and PM2 + PM3 + PP3 + PP4.

### 3.2 Literature review

Two publications were retrieved from the PubMed database, reporting seven patients with bi-allelic pathogenic variants in the TUBGCP2 gene from five families (7) (2). Table 1 provides an overview of the clinical data of the eight patients (seven previous patients and our patient), all of whom carry TUBGCP2 variants. TUBGCP2 pathogenic variants are summarized with a schematic diagram in the Figure S1. All eight patients (three females and five males, ages ranging from 1 to 10 years) presented with LIS and microcephaly. Additionally, five patients were born in consanguineous families, whereas three were in non-consanguineous families. All eight patients exhibited DD, with six (75%) having speech and motor delay, one (12.5%) experiencing speech delay, and one (12.5%) encountering motor delay. Seizures occurred in seven of the eight (87.5%) patients, with the age at seizure onset ranging from 5 months to 4 years and a median age of 21.5 months. Among the seven patients with seizures, five (71.4%) had abnormal EEG results, illustrating continuous slow background activity and epileptiform discharges. Furthermore, seven patients (87.5%) had dysmorphic facial features, including recruitment in the ear of five (71.4%); narrow forehead, thick eyebrows, and smooth philtrum in four each (57.1%); and bulbous nose, widened and separated teeth, and micrognathia in three each (42.9%). All eight patients had abnormal brain MRI findings, including pachygyria in eight (100%); thin corpus callosum in six (75%); cerebellar volume loss in three (37.5%); SBH, subependymal cyst, thin brainstem, and cystic foci in white matter in two each (25%); delayed myelination in one (12.5%); and periventricular leukomalacia in one (12.5%). In terms of the TUBGCP2 variants among the eight patients, six (75%) had missense variants, one (12.5%) had compound heterozygous mutations comprising one missense variant and one splice variant, and one (12.5%) had compound variants consisting of two missense variants and one deletion variant.

**Table 1:** Summary of cases with TUBGCP2 variants.

	Family 1 case 1	Family 1 case 2	Family 2 case 3	Family 2 case 4	Family 3 case 5	Family 4 case 6	Family 5 case 7	Our case
Countries	Turkey	Turkey	Turkey	Turkey	India	Iran	Poland	China
Sex	Female	Male	Male	Male	Female	Male	Male	Female
Age	10 yo	6 yo	6 yo	7 yo	1 yo	4 yo	8 yo	6 yo
Consanguinity	Yes	Yes	Yes	Yes	No	Yes	No	No
OFC/cm (Z score)	NA	NA	44.5	44	NA	34	46	46
Developmental delay	Motor + speech	Motor	Motor + speech	Speech	Motor + speech	Motor + speech	Motor + speech	Motor + speech
Autistic features	No	No	Yes	No	No	No	No	No
Facial features	Narrow forehead, thick eyebrows, prominent ear, bulbous nose, separated teeth, retrognathia	NA	Narrow forehead, thic eyebrows, upslanting palpebral fissures, bulbous nose, prominent ear, smooth philtrum, widely spaced teeth	Narrow forehead, bulbous nose, prominent ear, smooth philtrum, retrognathia	Short and sloped forehead, thick eyebrows, puffy eyelids, full lips, retromicrognathia	Bitemporal narrowing, upslanting palpebral fissure, micrognathia, midface hypoplasia, prominent ears and lips	Smooth philtrum, prominent ears	Narrow forehead, thick eyebrows, bulbous nose, smooth philtrum, widely spaced teeth
Tone	Hypotonia	Contractures, spasticity	Truncal hypotonia	Normal	Truncal hypotonia spasticity	Truncal hypotonia	Normal	Normal
Reflex	hyperreflexia	hyperreflexia	NA	NA	Hyperreflexia	NA	Normal	Normal
Ophthalmology	NA	NA	Myopia	NA	Cortical blindness	Optic atrophy	Myopia, astigmatism	Normal
MRI	Pachygyria, cerebral and cerebellar atrophy, cystic foci in white matter, and thin corpus callosum	Pachygyria, cerebral and cerebellar atrophy, decreased white matter volumes, cystic foci at the centrum semiovale and thin corpus callosum	Pachygyria, thin corpus callosum, especially in the posterior region, cerebellar atrophy	Posterior dominant pachygyria	Pachygyria, SBH, delayed myelination,subependymal cyst,thin corpus callosum and brainstem	Pachygyria, SBH, hyperintense periventricular white matter, subependymal cyst, thin corpus callosum and brainstem	Pachygyria in the temporal lobes and thin corpus callosum	Posterior dominant pachygyria
Seizures: onset/type	6 months intractable epilepsy	3 years intractable epilepsy	6 years, 9 months generalized	no	5 months generalized	7 months/generalized	NA/generalized	4 years/generalized
EEG	NA	NA	Continuous slow background activity and frequent multifocal epileptiform discharges (6.5 years)	NA	Abnormal	Frequent epileptiform discharges	Paroxysmal epileptiform activity localized to central area	Sharp slow waves and spike slow waves
Genetic test								
Position (hg19)/Nucleotide (Protein)/Variants	NA/c.1015 G>A (p.Glu311Lys)/homozygous	NA/c.1015 G>A (p.Glu311Lys)/homozygous	Chr10: 135106570:C>T/c.997C>T(p.Arg333Cys)/homozygousmi missense variant	Chr10: 135106570:C>T/c.997C>T(p.Arg333Cys)/homozygousmi missense variant	Chr10: 135099012:G>C/c.1843G>C(p.Ala615Pro)/homozygousmi missense variant	Chr10: 135099012:G>C/c.1843G>C(p.Ala615Pro)/homozygous	Chr10: 135106678:C>T/c.889C>T(p.Arg297Cys)/missense variant/Chr10: 135097508:A>G/c.2025-2A>G/splice variant	Chr10:135098588-135116445/LOH exon:2-14 (17858 bp (NM_00125 6617)/deletion variant Chr10:135113590:C>T/c.178 C>T (p.P60S(p.Pro60Ser) (NM_001256617)/missense variant Chr10:135111534:T>C/c.538T>C(p.W180R(p.Trp180Arg)(NM_001256617)/missense variant

yo, years old; OFC: occipital frontal circumference; subcortical band heterotopia: SBH; EEG, electroencephalograph; MRI, magnetic resonance imaging; NA, not available.

## 4 Discussion

**Figure 6:**
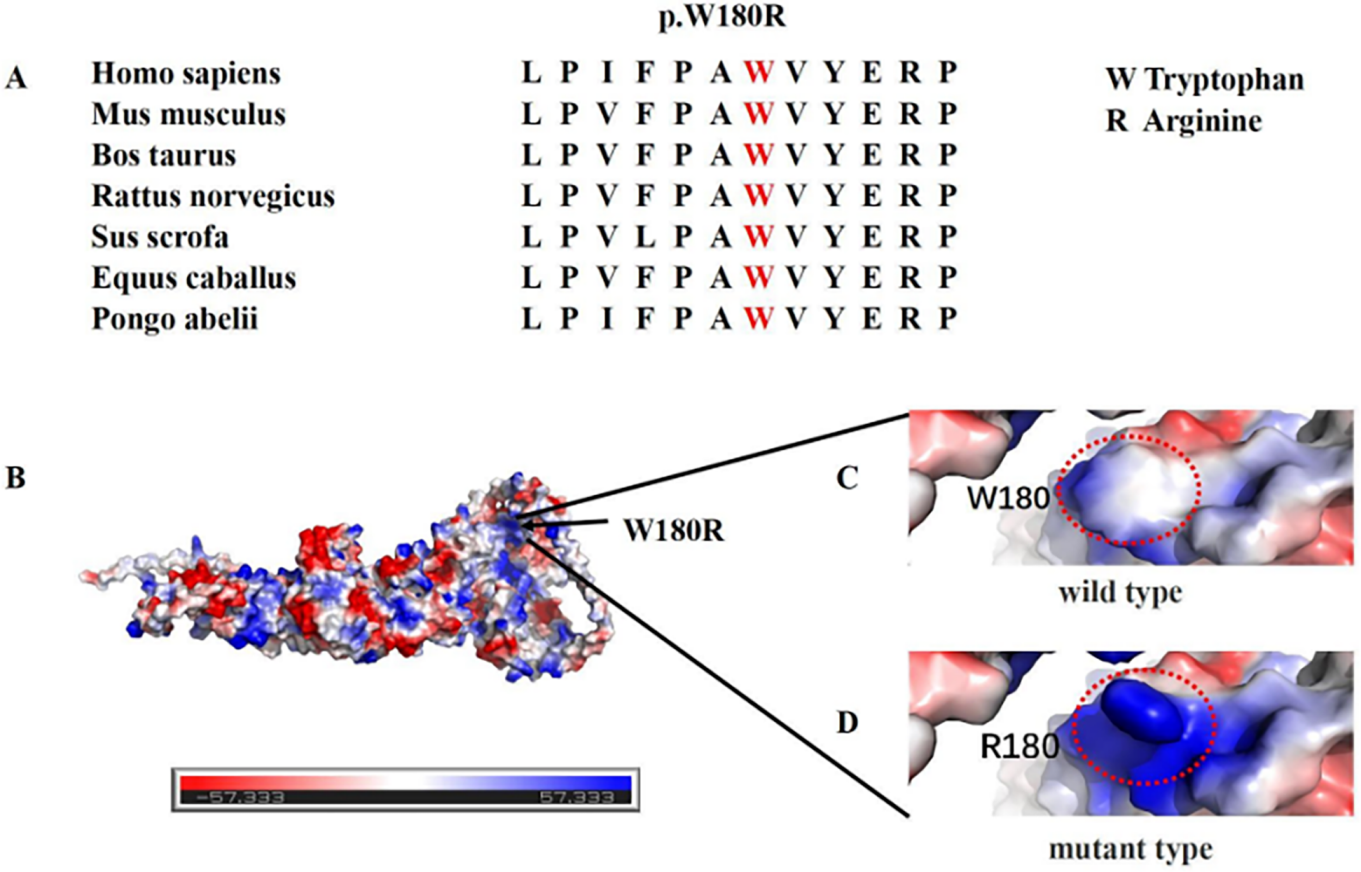
**(A)** Multiple sequence alignment analysis revealed high conservation of p.W180R across different species. **(B)** Electrostatic potential diagram of TUBGCP2, with negative and positive charges shown in red and blue, respectively. **(C,D)** 3D structural analysis of the p.W180R protein further suggested that the mutated p.W180R may disrupt the electrostatic interaction of the proteins. Software: PyMOL PBD: AF-Q9BSJ2-F1.

LIS displays significant genetic heterogeneity, with many variant genes encoding proteins related to MT structure or function. MTs are crucial for cortical development and neuronal migration. Consequently, variants in the genes encoding alpha-tubulin (ɑ-tubulin; TUBA1A), beta-tubulin (β-tubulin; TUBB2A, TUBB2B, TUBB3, TUBB4A, and TUBB), and gamma-tubulin (γ-tubulin; TUBG1) may affect MT function, leading to brain malformations including LIS, schizencephaly, polymicrocerebellar disease, cerebellar disease, and simplified cyclotorsion (8). The TUBGCP2 gene encodes the γ-tubulin complex 2 protein (GCP2). GCP2, together with GCP3, GCP4, GCP5, and GCP6, forms the γ-tubulin small complex (γ-TuSC), a critical component of the MT-organizing centers. Considering that the TUBGCP2-encoded GCP2 protein is a core element of γ-TuSC (a template for MT formation), Mitani et al. have proposed TUBGCP2 as a candidate tubulinopathy-associated gene (2). The TUBGCP2 variants (c.178 C>T, c.538T>C, and LOH exon: 2–14) reported in our study are novel variants that have never been reported, thus expanding the genotype of LIS spectrum disorders associated with TUBGCP2 variants. In the current study, we identified compound heterozygous variants consisting of missense variants and an exon deletion variant in TUBGCP2. Furthermore, multi-hazard prediction software analysis indicated that p.W180R was more deleterious. Additionally, multiple sequence alignment analysis revealed high conservation of p.W180R across different species (Figure 6). 3D structural analysis of the p.W180R protein further suggested that the mutated p.W180R replaced the nonpolar tryptophan with the positively charged arginine compared to the WT (wild type) model (Figure 6), indicating structural changes in the protein with potential disruption of the electrostatic interaction of the proteins. Currently, functional and proteomic studies on TUBGCP2 gene variants in human neurons are lacking. Nevertheless, Gungor et al. have employed proteomics to investigate the functional effect of TUBGCP2 variants in fibroblasts. Their results have suggested that these variants may disrupt the electrostatic interaction between GCP2 and GCP3, resulting in a slight delocalization of γ-tubulin during the cell cycle. This alteration may also lead to the dysregulation of the proteins involved in cellular adhesion to the extracellular matrix, a vital process for cell migration and invasion (7). Fibroblasts have been established as a suitable model for studying the molecular etiology of neurological diseases. Therefore, we speculated that TUBGCP2 variants in neurons could cause LIS by disrupting the electrostatic interaction of proteins, ultimately affecting MT function in cell migration. Apart from their role in cell migration, MTs are also critically involved in the myelination and remyelination stages of the central nervous system, consistent with the detection of leukopenia and myelin dysplasia in some patients (7). Future research incorporating functional and proteomic evaluation of TUBGCP2 gene variants in human neurons may help elucidate the pathophysiology of this rare disease and explore potential therapeutic avenues.

Variants in different tubulin genes induce various phenotype and they might alter the dynamics properties of microtubule polymers in several ways. The first tubulin gene associated with impaired cerebral cortical development was TUBA1A, a key gene regulating the synthesis of *α*-tubulin, and its variants affect the folding of tubulin heterodimers and the interaction of microtubule-binding proteins, resulting in microtubule dysfunction and neuronal progenitor cell motility defects, which are related to lissencephaly combined with cerebellar atrophy (9). TUBG1 is one of two human γ-tubulin genes, the features of TUBG1 variants resemble LIS spectrum diseases (10). Both ɑ-tubulin and γ-tubulin gene variants predominantly lead to LIS spectrum diseases, whereas β-tubulin gene variants may result in a normal cortical pattern (8, 11). This may be due to the distinct role of β-tubulin. Gamma-complex component genes (TUBGCP4, TUBGCP5, and TUBGCP6) are critical in neuronal progenitor proliferation, and its variants cause cell proliferation defects and increased apoptosis, which have been reported to be associated with microcephaly (2). Li et al, generated the zebrafish tubgcp3 mutants using the CRISPR/Cas9 system and found that the zebrafish embryos also exhibit the microcephaly phenotype (12). However, the phenotypes attributed to TUBGCP2 variants have not yet been determined (11). Nevertheless, previous studies have demonstrated that the pathogenic TUBGCP2 variants lead to autosomal recessive neurodevelopmental disorder traits consisting of microcephaly, dysmorphic facial features (such as a characteristic narrow forehead, thick eyebrows, bulbous nose, prominent ears, and widened and separated teeth), and DD (including speech and motor delays), with or without seizures. Moreover, a few patients present with visual system abnormalities, including optic atrophy and cortical blindness. This observation may be ascribed to the varying degrees of TUBGCP2 expression in other tissues, such as the optic nerve, highlighting that altered TUBGCP2 expression is not limited to its high expression in the brain (2).

Brain MRI findings indicate that TUBGCP2-related LIS spectrum disorders exhibit distinctive neuroradiological features, including microcephaly and LIS (agyria, pachygyria, and SBH). These observations are indicative of impaired neuronal migration. In particular, the posterior brain region showed more severe abnormalities than the anterior region in three (37.5%) patients, suggesting that TUBGCP2-associated LIS may manifest a posterior (P) >anterior (A) gradient. This finding has also been observed in TUBA1A-related LIS. Moreover, the P>A gradient may be associated with various structural abnormalities, including cerebellar hypoplasia, basal ganglia malformation, thin or absent corpus callosum, congenital microcephaly, ventricular dilatation, and hippocampal and brainstem abnormalities (13). Further, comparisons between LIS caused by LIS1 and DCX variants revealed that LIS1 variants were more severe in the parietal and occipital lobes, while DCX variants showed greater severity in the frontal lobe, suggesting an A>*P* gradient for DCX-associated LIS (14). This result suggests that certain differences exist between LIS caused by the gene variants encoding proteins related to MT structure (LIS1 and DCX) and function (TUBGCP2 and TUBA1A). MRI is the primary investigative modality for assessing patients with LIS, facilitating the identification of the radiological features (e.g., severity gradient, cortical thickness, and other structural abnormalities) to prioritize genetic testing (15). For example, the priority for genetic testing would be TUBGCP2 and TUBA1A variants in LIS with a P>A gradient, while the genetic testing would be prioritized for DCX variants in ILS with an A>P gradient. Additionally, a few patients present with noncortical malformations, including thin brainstem and corpus callosum, cerebellar atrophy, and SBH (Table 1). These malformations are commonly observed in patients with tubulin disease, indicating the usefulness of these diagnostic indicators for detecting tubulin gene involvement (13).

Genetic tests are required to be quickly completed to confirm the diagnosis of patients with dysmorphic facial features, microcephaly, and DD. The advancement of molecular genetics has led to the detection of an increasing number of genetic variants associated with LIS, indicating the significant genetic heterogeneity of LIS. Although most LIS-associated genes result in dominant phenotypes via mono-allelic variants, TUBGCP2 induces recessive traits through bi-allelic variants (2). In our patient, missense variants were the primary TUBGCP2 variant type. The compound variants consisting of missense and deletion mutants (c.178 C>T, c.538T>C, and LOH exon: 2–14) are novel variants that have been reported for the first time in this study. Furthermore, the clinical phenotype of the patient was found to be consistent with the genotype, and we speculated that the variant was pathogenic based on the ACMG guidelines. Nonetheless, further research studies and case reports are warranted to confirm our findings. Moreover, identifying brain development-related genes and exploring their function could lead to a better understanding of the relevant molecular mechanisms and provide potential therapeutic intervention for neurodevelopmental disorders. Although gene-specific treatments for LIS are currently scarce, an animal model study by Manent et al. suggested the potential of renewing neuronal migration by re-expressing the missing genes postnatally (16).

## 5 Conclusions

In summary, our study revealed that LIS has significant genetic heterogeneity. This study presents the first reported case of a patient with TUBGCP2-associated LIS in China based on trio-WES analysis. Furthermore, we identified novel compound variants comprising missense and deletion mutants in TUBGCP2, thereby expanding the genotype of TUBGCP2-related LIS spectrum disorders. However, the limited data in our study makes it challenging to establish a specific genotype-to-phenotype correlation. Thus, future case reports should perform genotype-phenotype analyses and functional investigation of LIS associated with TUBGCP2 gene variants.

## Data Availability

The original contributions presented in the study are included in the article/Supplementary Material, further inquiries can be directed to the corresponding author/s.
