# Supplementary material for: TUBGCP2 variants cause lissencephaly spectrum disorders: a case report and literature review
Source: Front Pediatr. 2025 Feb 13;13:1476390. doi: 10.3389/fped.2025.1476390 (PMC11866843; doi:10.3389/fped.2025.1476390)
Supplement: Supplementary file 1 [file Table1.docx]

**Table S1. Primer sequence of Sanger sequencing**

| **Primer name** | **Primer sequence** | **Product length** |
| --- | --- | --- |
| TUBGCP2-538-F | CCAGAAATAAACACATGACATACCT | 364bp |
| TUBGCP2-538-R | ATTGAGTAATCATCTTTGCCGTAG |  |
| TUBGCP2-178-F | CACATGCGGGAAGCTCAAAG | 454bp |
| TUBGCP2-178-R | AATTCCATTGGCTTATTGTGTTCA |  |
