# Supplementary material for: TUBGCP2 variants cause lissencephaly spectrum disorders: a case report and literature review
Source: Front Pediatr. 2025 Feb 13;13:1476390. doi: 10.3389/fped.2025.1476390 (PMC11866843; doi:10.3389/fped.2025.1476390)
Supplement: Supplementary file 2 [file Table2.docx]

**Table S2. Primer sequence of qPCR**

| **Primer name** | **Primer sequence** | **Product length** |
| --- | --- | --- |
| ALB-F | ACTCAGTGCACTTGTTGAGCTCGT | 114bp |
| ALB-R | TCGTCAGCCTTGCAGCACTTCT |  |
| TUBGCP2-exon2-F | CAGTGGTAGTGACGTACGGGGTC | 97bp |
| TUBGCP2-exon2-R | GAACTGCTTAGCCTGCTGCGT |  |
| TUBGCP2-exon8-F | ACCGAGGGAGGCCAGGATGTC | 176bp |
| TUBGCP2-exon8-R | AGTACGGGCAGGTGAACCACG |  |
| TUBGCP2-exon14-F | CGAGTGCTGCTTGGCGGTTT | 132bp |
| TUBGCP2-exon14-R | GAGGTGCCGCCACGTCTCT |  |

Quantitative Real-time PCR:qPCR
