# Supplementary figures and images for: TUBGCP2 variants cause lissencephaly spectrum disorders: a case report and literature review
Source: Front Pediatr. 2025 Feb 13;13:1476390. doi: 10.3389/fped.2025.1476390 (PMC11866843; doi:10.3389/fped.2025.1476390)

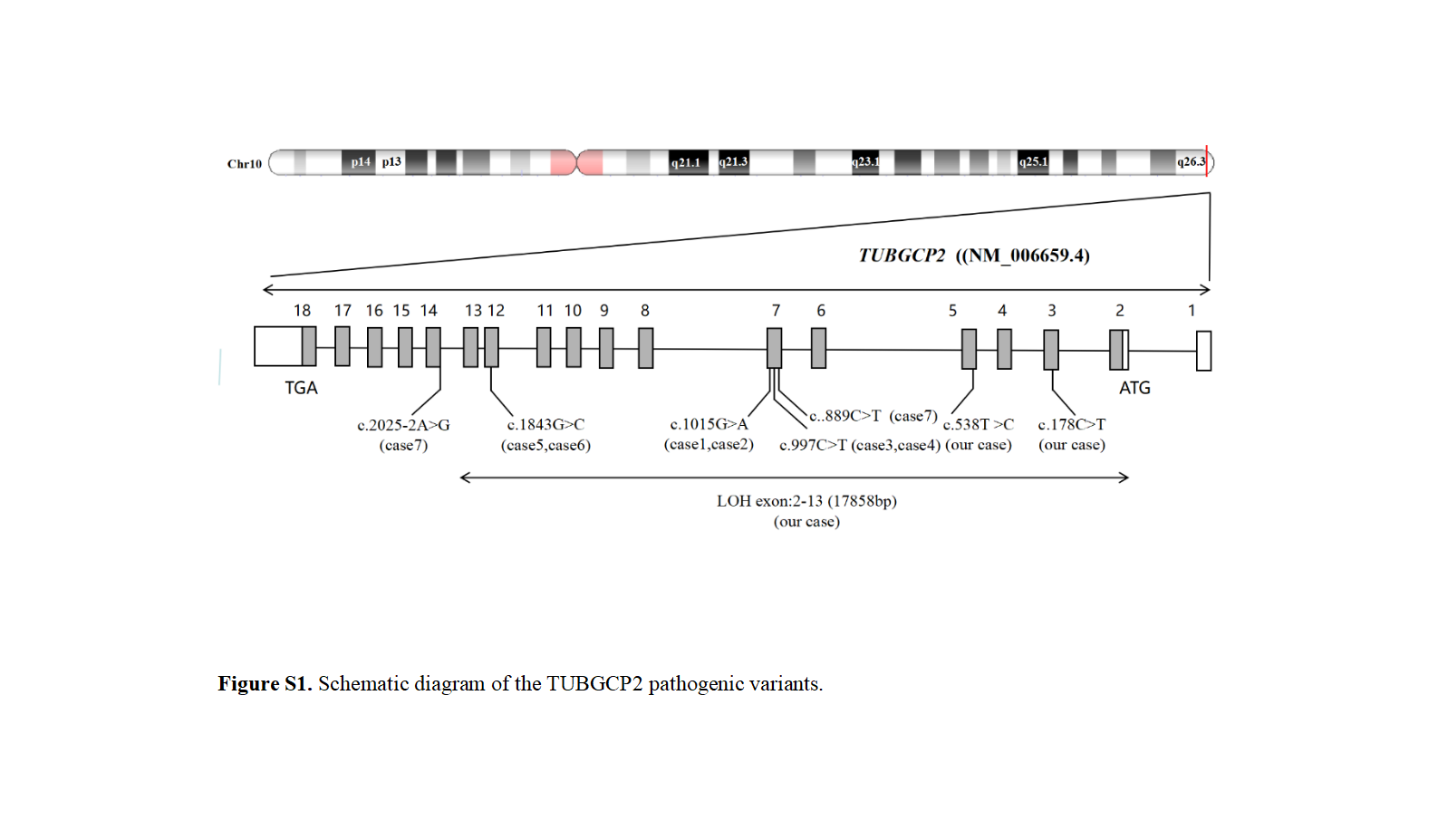

Supplement: Supplementary file 3 [file Image1.tif]
